# Pitfalls and diagnostic challenges in TRECS-based newborn screening for Artemis, FOXN1 and Moesin deficiencies

**DOI:** 10.3389/fimmu.2026.1947649

**Published:** 2026-10-01

**Authors:** Carlota C. Ruigómez-Martín, Celia Nieto-López, Lucía Dueñas-Prieto, Juan D. Acevedo-Ávila, Francisco J. Gil-Etayo, Inés Esteban-Díez, Cristina García-Muro, Juan F. Quesada-Espinosa, María J. Díaz-Madroñero, Leyre Echeverría-Sádaba, Samuel Martín-Rodríguez, Elena García-González, Marta González-Vicent, Iván Bernardo, Carmen Rodríguez-Vigil Iturrate, José T. Ramos-Amador, Marta Fernández-Gaitán, Nerea Salmón-Rodríguez, Luis I. González-Granado, Luis M. Allende

**Affiliations:** 1 Department of Immunology, Hospital Universitario 12 de Octubre, Madrid, Spain; 2 Instituto de Investigación Sanitaria Hospital 12 Octubre (imas12), Madrid, Spain; 3 Department of Hematology and Instituto de Investigación Biomédica de Salamanca (IBSAL), Hospital Universitario de Salamanca, Salamanca, Spain; 4 Department of Peadriatics, Hospital Universitario San Pedro, Logroño, Spain; 5 Department of Genetics, Hospital Universitario 12 de Octubre, Madrid, Spain; 6 Dysmorphology and Genetics Unit (UDisGen), Hospital Universitario 12 de Octubre, Madrid, Spain; 7 Genetics Section, Department of Clinical Analysis, Hospital Universitario San Pedro, Logroño, Spain; 8 Newborn Screening Unit, Department of Clinical Chemistry, Hospital Universitario Miguel Servet, Zaragoza, Spain; 9 Hematopoietic Stem Cell Transplantation Unit, Hospital Universitario Niño Jesus, Madrid, Spain; 10 Department of Clinical Analysis, Hospital Universitario San Pedro, Logroño, Spain; 11 Department of Hemato-Oncology Peadriatics, Hospital Universitario Miguel Servet, Zaragoza, Spain; 12 Department of Peadriatics, Hospital Universitario 12 de Octubre, Madrid, Spain; 13 School of Medicine, Complutense University of Madrid, Madrid, Spain

**Keywords:** Artemis, FOXN1, inborn errors of immunity, Moesin, newborn screening, severe combined immunodeficiency, thymopoiesis, TRECs

## Abstract

Newborn screening (NBS) based on T-cell receptor excision circles (TRECs) has revolutionized the early diagnosis of severe combined immunodeficiency (SCID), facilitating the identification of affected newborns before the onset of life-threatening infections. However, reduced TRECs levels are a surrogate marker of impaired thymic output rather than a disease-specific finding. Accordingly, low values can result from highly heterogeneous and distinct biological mechanisms of T-cell ontogeny that extend beyond classical SCID phenotypes. This study describes three patients with inborn errors of immunity (IEI) identified through abnormal TRECs screening who were subsequently diagnosed with Artemis, FOXN1, and Moesin deficiencies. These clinical cases demonstrate that primary T-cell immunodeficiencies can arise from defects at distinct biological levels of T-cell development, specifically in impaired antigen receptor V(D)J recombination (characteristic of NHEJ deficiencies and other SCID conditions), disrupted thymic stromal microenvironment function and compromised peripheral lymphocyte homeostasis, respectively. Consequently, optimizing clinical outcomes after a positive newborn screening requires the systematic integration of clinical, immunological and molecular findings. This diagnostic approach should involve specialized immunodeficiency centers with access to advanced flow cytometry techniques and molecular confirmation to ensure an accurate and definitive diagnosis. Ultimately, therapeutic decisions from supportive care to definitive hematopoietic stem cell transplantation or gene/enzymatic therapy for SCID patients must be guided by the precise underlying pathogenic mechanism and the specific molecular defect.

## Introduction

Inborn errors of immunity (IEI) that impair T-cell development and function comprise a clinically heterogeneous group of potentially life-threatening disorders caused by defects at multiple levels of lymphocyte ontogeny. These conditions can affect early lymphocyte commitment (cytokine signaling, antigen receptor recombination and DNA repair), thymic stromal microenvironment, as well as alterations of lymphocyte homeostasis (1–4). Severe combined immunodeficiency (SCID) represents the most profound manifestation within this spectrum. It is characterized by severe impairment of adaptive immunity resulting increased susceptibility to infections, immune dysregulation, failure to thrive and high mortality during infancy in the absence of early therapeutic intervention (5, 6).

The implementation of newborn screening (NBS) based on quantification of T-cell receptor excision circles (TRECs) has transformed the early detection of SCID and other non-SCID T-cell lymphopenia, over the past decade (7–10). TRECs are episomal DNA molecules generated as by-products of somatic V(D)J recombination during thymocyte development and serve as surrogate markers of thymic output. Since these circular DNA fragments do not replicate during cell division, their quantification in peripheral blood provides an indirect estimate of recent thymic emigrants and *de novo* T-cell production (9, 11). This strategy enables identification of neonates with impaired T-cell ontogeny before the onset of severe infectious complications, significantly improving clinical outcomes through timely therapeutic intervention (7, 9, 12).

TREC-based NBS was primarily implemented for the early detection of severe combined immunodeficiency (SCID) (7–10). However, TRECs are biomarkers of thymic production of newly generated T cells rather than disease-specific markers (7, 9, 11). Consequently, low or absent TRECs identify not only infants with SCID but also a broad spectrum of non-SCID T-cell lymphopenias (7, 9, 13, 14).

SCID results from severe impairment of T-cell differentiation and comprises typical and leaky or atypical forms (15). In contrast, non-SCID T-cell lymphopenia includes thymic developmental or stromal disorders, such as FOXN1 deficiency and syndromic thymic hypoplasia, other inborn errors of immunity associated with impaired T-cell production, secondary T-cell lymphopenia related to conditions such as prematurity or lymphatic loss and idiopathic forms (7, 9, 13, 14). Thus, an abnormal TREC newborn screening result identifies infants with impaired T-cell output who require further immunological and genetic evaluation to distinguish SCID from non-SCID causes of T-cell lymphopenia, but do not identify the underlying etiology responsible for this defect (13–15).

Mechanistically, reduced thymopoiesis related to SCID (15) arises from several primary inherited defects affecting distinct levels of T-cell ontogeny: (i) cytokine signaling defects (16, 17) (common γ−chain, Jak3, IL-7Rα) and metabolic toxicity (ADA) (18); (ii) abnormalities in antigen receptor recombination (RAG) (19), DNA repair machinery (Artemis and other radiosensitive-SCID conditions) (20) and pre- and post-TCR checkpoint arrest (CD3 and CD45 deficiencies, respectively) (21, 22). Similarly, impaired thymopoiesis related to non-SCID disorders with T-cell lymphopenia can arise from varied molecular and cellular mechanisms underlying congenital or acquired immunodeficiencies (4, 15). Although these defects involve different biological compartments, they ultimately converge on a common measurable outcome, a reduced thymic export of naïve T cells and, consequently, decreased TRECs production. Thus, abnormal TRECs results should not be equated with SCID itself but rather as evidence of T-cell lymphopenia secondary to heterogeneous biological processes. Therefore, abnormal NBS results reflect fundamentally different pathogenic mechanisms with distinct diagnostic, prognostic, and therapeutic implications. Defining the biological basis of an abnormal TRECs result is essential for accurate diagnosis and appropriate clinical management (4, 7, 9, 13, 14).

The SCID and CID conditions explored in this work illustrate how distinct biological mechanisms, as V(D)J recombination and DNA repair (Artemis deficiency), thymic stromal development (FOXN1) and immune-related actinopathies (Moesin) can affect the production of naïve T-lymphocyte production and TRECs. Ultimately, they highlight that TRECs quantification reflects a general biological output rather than a disease-specific diagnosis. Hence, this study aims to explore the clinical pitfalls and interpretive challenges associated with NBS based on TRECs and subsequent genetic evaluation using NGS and Sanger technologies, with emphasis on the need for a broader clinical, immunological and molecular framework.

## Material and methods

### NBS and TRECs

The study population reflects data from the SCID NBS programs in the Autonomous Communities of La Rioja, Aragón and Navarra (October 2025 – August 2026), covering approximately 12,388 screened newborns.

TRECs copy numbers were quantified in dried blood spots (DBS, punches of 3.2 mm) or DNA samples using real-time quantitative polymerase chain reaction (RT-qPCR). Reactions were performed with a commercial kit (LightMix^®^ Kit TREC SMA Newborn Screening, Roche Diagnostics, Mannheim, Germany) and run in a Light Cycler 480 II from Roche Diagnostics.

### Immunophenotype and humoral studies

Immunophenotyping was performed on whole peripheral blood for assessment of T, B and NK cells. The T-cell compartment was evaluated based on CD45RA and CCR7 expression in CD4+ and CD8+ T cells, defining naïve (CD45RA+CCR7+), central memory (CD45RA−CCR7+), effector memory (CD45RA−CCR7−), and TEMRA (CD45RA+CCR7−) T-cell subsets. Recent thymic emigrants (RTE) were informed as CD4^+^CD31^+^CD45RA^+^ lymphocytes. Cell acquisition was conducted using AQUIOS and DxFLEX flow cytometers (Beckman Coulter, Miami, FL, USA) and data were analyzed with Kaluza software version 2.1 (BeckmanCoulter).

Serum total immunoglobulin concentrations (IgG, IgA and IgM) were determined by nephelometry on an IMMAGE 800 Immunochemistry System (Beckman Coulter), according to the manufacturer’s instructions.

### Next-generation sequencing

Genomic DNA (gDNA) was extracted from EDTA-anticoagulated peripheral blood using the QIAamp DNA Mini Kit (Qiagen, Heidelberg, Germany) and quantified using the Qubit dsDNA HS Assay Kit (Thermo Fisher Scientific, Waltham, MA, USA). Molecular screening was performed using three different next-generation sequencing (NGS) approaches. For the amplicon-based strategy, sequencing was performed on an Ion Torrent PGM platform (Thermo Fisher Scientific, Waltham, Massachusetts, USA) using a custom targeted panel of 192 genes involved in IEIs (Ampliseq, Thermo Fisher Scientific). Libraries were prepared with 20 ng of DNA to generate 300-bp fragments, followed by emulsion PCR and sequencing according to standard manufacturer protocols. For the targeted hybrid-capture approach, a custom targeted panel of 476 genes associated with IEIs (SureDesign; Agilent Technologies, Santa Clara, CA, USA) was executed on the MiSeq platform (Illumina, San Diego, CA, USA), generating 75 bp paired-end reads with a mean depth of 100X and >98% coverage. Finally, for the whole-exome sequencing (WES), target enrichment was performed using Clinical Research Exoma (CRE) V4 design (Agilent Technologies) and sequenced on a DNBSEQ-G400 platform (MGI Tech, Shenzhen, China) to generate paired-end reads with a mean depth of 100X and >98% coverage.

All generated data were aligned to the human GRCh37 (hg19) reference genome. Variant calling was performed using Torrent Suite Software (version 5.12) for the amplicon-based data, the Alissa Reporter and Interpret software (Agilent Technologies) for the hybrid-capture data, and the Geneyx software leveraging the DRAGEN v3 pipeline (Illumina) for the WES dataset. Variant pathogenicity was assessed according to the update American College of Medical Genetics and Genomics (ACMG) guidelines (23),considering allele frequency, *in silico* predictors (SIFT, PolyPhen-2, CADD), immunological and functional data, familial segregation, and clinical phenotype. Candidate variants were visually inspected using the Integrated Genomics Viewer (IGV) (24).

### Sanger sequencing and primer design

All candidate germline variants identified by NGS were validated by Sanger sequencing and familial segregation were informed. In the case of Artemis deficient patient, the presence of a highly homologous pseudogene for the *DCLRE1C* gene, specific primers were designed to ensure exclusive amplification of the functional locus. The pathogenic variant in *DCLRE1C* was confirmed using the following exon-specific primers:5´ cgggaggtaggaggtgat (forward) and 5´ acacccagatgataaccccg (reverse).

## Results

The NBS program of 3 Autonomous Communities of Spain tested 12,388 newborns in 11 months. 22 (0.18%) had an initially abnormal TRECs result. All were re-tested using the same sample, 9 remained abnormal, whereas 13 normalized TRECs results. Of the 9 infants with persistently abnormal results, 2 died before a new sample could be obtained. The re-sampling of the remaining 7 infants, 5 subsequently normalized their TREC levels, while 2 continued to show absent or low TRECs. Furthermore, 2 infants were referred for immunological assessment. Final diagnoses included no cases of SCID, 1 case of non-SCID T-cell lymphopenia, 1 case of thymic/stromal defect, no genetically unresolved cases, and 5 cases of transient or secondary TRECs abnormalities, including prematurity. Patients 2 and 3 in the present series were identified through NBS and were subsequently diagnosed with FOXN1 and Moesin deficiencies, respectively.

To evaluate the diverse pathophysiological mechanisms underlying an abnormal TRECs result (**Table 1**), we selected three illustrative cases characterized by significant diagnostic pitfalls and interpretative challenges: (i) a case of Artemis deficiency (*DCLRE1C*) where underlying pseudogene interference masked the true homozygous state of the pathogenic variant; (ii) a case of monoallelic FOXN1 deficiency (*FOXN1*) whose clinical phenotype initially mimicked classical SCID but ultimately reflected primary thymic stromal dysfunction; and (iii) a case of Moesin deficiency (*MSN*) presenting as leaky SCID, characterized by abnormal TRECs and severe lymphopenia despite preservation of naïve T-cell populations. The criteria for referral patients were according to Argudo-Ramirez et al. and De Felipe et al. (25, 26) (**Supplementary Figure 1**). These cases underscore the need to interpret TRECs results within a broader clinical and immunological context (**Figure 1**), taking into account the main conditions associated with low TRECS levels (**Table 1**; **Supplementary Figure 2**).

**Table 1:** Conditions associated with low or absent TRECs in NBS: SCID versus non-SCID T-cell lymphopenia*.

Diagnostic category after abnormal trec result	Mechanistic group	Representative causes
SCID	Typical SCID, leaky/atypical SCID and Omenn syndrome (intrinsic hematopoietic T-cell developmental defects)	IL2RG, JAK3, IL7R, RAG1/RAG2, DCLRE1C (Artemis), LIG4, NHEJ1, PRKDC, ADA, AK2 and other established SCID genotypes. Selected rare IEI genotypes (e.g., RMRP, RAC2, BCL11B or MSN) may also meet leaky or atypical SCID criteria in severe presentations. Represented by Patient 1 with biallelic DCLRE1C deficiency.
NON-SCID T-CELL LYMPHOPENIA	Thymic development/thymic stromal disorders	22q11.2 deletion syndrome or DiGeorge spectrum, CHARGE syndrome (CHD7), FOXN1 deficiency, PAX1-associated thymic defect and other congenital athymia. Represented by Patient 2 with monoallelic LOF FOXN1 deficiency.**
	Genetic syndromes and other IEI/CID associated with T-cell impairment or abnormal T-cell homeostasis	Trisomy 21, trisomy 18, Noonan syndrome, Kabuki syndrome, ataxia-telangiectasia (ATM), Nijmegen breakage syndrome (NBN), RECQL4-related disorders; combined immunodeficiencies such as DOCK8 or EXTL3 deficiency when SCID criteria are not fulfilled. Represented by Patient 3 with monoallelic MSN deficiency.
	Idiopathic T-cell lymphopenia	Persistent or transient T-cell lymphopenia with no identified genetic, syndromic or secondary cause after appropriate assessment.
SECONDARY/REVERSIBLE T-CELL LYMPHOPENIA	Prematurity or low birth weight	Prematurity, very low birth weight and physiologic immaturity of thymic output.
	Lymphocyte loss, congenital disease or critical illness	Chylothorax or chylous loss, hydrops or third-space losses, congenital heart disease, congenital diaphragmatic or gastrointestinal anomalies, multiple congenital anomalies, asphyxia or severe neonatal disease.
	Infection or drug-related suppression	Severe neonatal infection/sepsis; maternal immunosuppressive treatment; neonatal corticosteroids, chemotherapy or immunosuppressive treatment.
OTHER SCREENING OUTCOME	Abnormal TREC result without confirmed T-cell lymphopenia	Normal T-cell subsets on confirmatory flow cytometry, transient isolated low TRECs, or pre-analytical/technical false-positive result.

*Should be distinguished from both SCID and non-SCID T-cell lymphopenia. The TREC assay is a screening test of thymic output, not a disease-specific diagnostic test. Low or absent TRECs may closely mimic SCID because impaired thymic output, but the primary defect is extrinsic to hematopoietic progenitors. **This distinction is critical because HSCT may not correct a primary thymic stromal defect.

**Figure 1:**
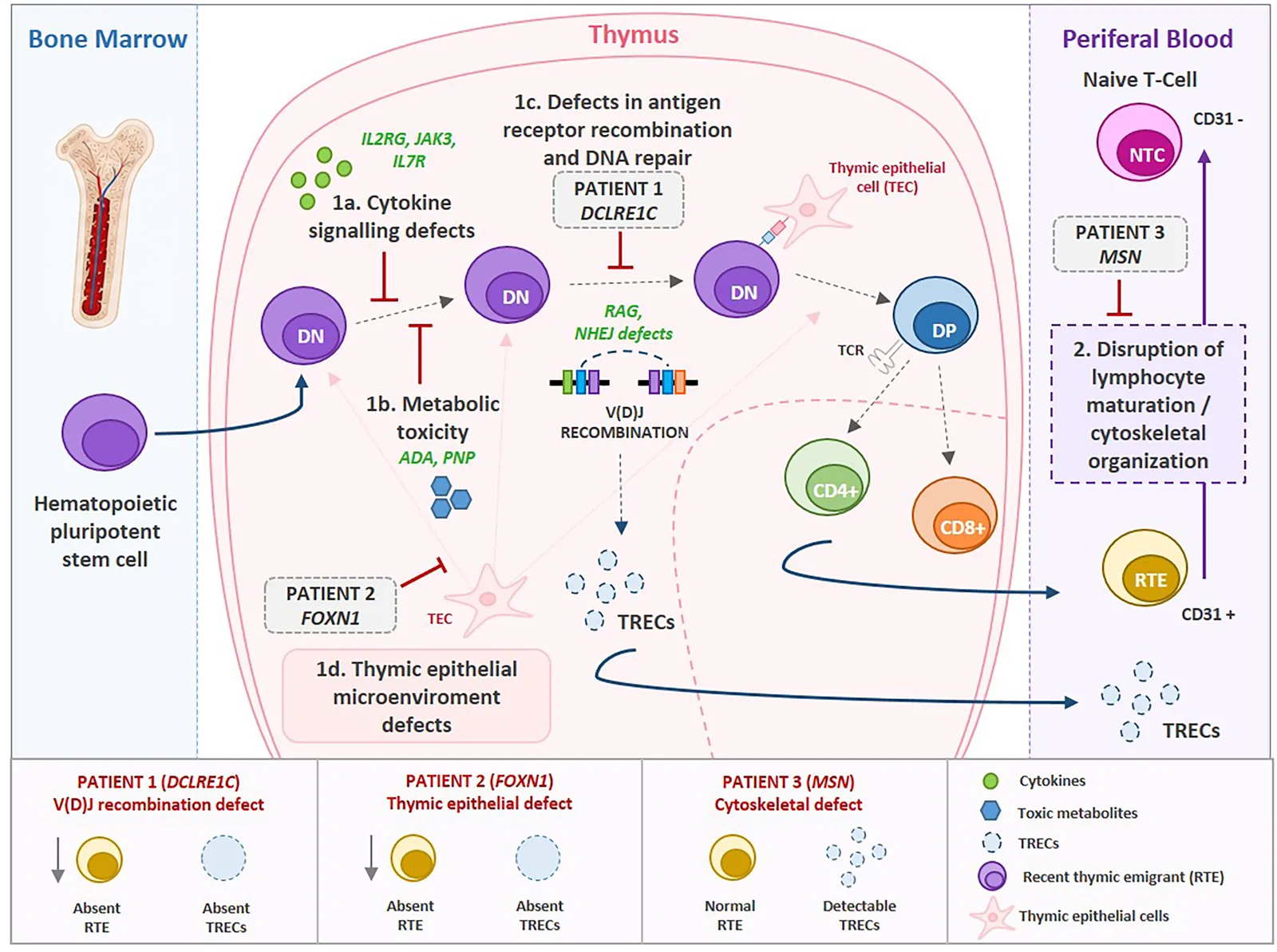
Mechanistic basis of abnormal TREC-based newborn screening in three representative inborn errors of immunity. Simplified representation of pathophysiological pathways that converge on reduced thymic output. Patient 1 (*DCLRE1C*) shows an intrinsic hematopoietic defect that affects lymphoid progenitors, specifically in DNA repair machinery, leading to absent TRECs and profound impairment of thymocyte development. Patient 2 (*FOXN1*) illustrates a defect within the thymic epithelial microenvironment, compromising stromal support for intrinsically normal lymphoid progenitors. Patient 3 (*MSN*) represents a disruption in lymphocyte maturation and cytoskeletal organization, where T-cell receptor recombination remains intact, but defects in peripheral lymphocyte homeostasis or trafficking lead to reduced peripheral TRECs despite preserved RTE. The lower panel summarizes the characteristic TREC and RTE blood profiles for each patient. Abbreviations: DN, double negative; DP, double positive; RTE, recent thymic emigrant; TEC, thymic epithelial cell; TREC, T-cell receptor excision circle.

### Case 1. Artemis deficiency

#### Clinical and immunological findings

The patient is a male infant referred at 3 weeks of age to rule out SCID. He was diagnosed before the implementation of routine NBS for SCID in our region. TRECs quantification was performed at admission (during the neonatal period), as part of the diagnostic evaluation. He presented with severe erythroderma, failure to thrive and a family history suggestive of an IEI. He showed absent TRECs (0/0 copies in a DNA sample), immunological evaluation revealed lymphopenia (1830 cells/µL) with a severe reduction of T lymphocytes (689 cells/µL) and an almost complete absence of B cells (1 cell/µL), whereas NK cells were preserved (977 cells/µL). T-cell compartment analysis demonstrated a profound reduction of thymic output, with absent recent thymic emigrants (RTEs) and an almost complete absence of naïve CD4^+^ and CD8^+^ T cells (0.3% and 0.2%, respectively) (**Figure 2**) together with a marked expansion of effector-memory CD4^+^ T cells (87.1%). Increased expression of HLA-DR was observed mainly in CD8^+^ T-cell subsets (65%). Humoral studies showed low IgG (305 mg/dL) (**Table 2**). Given the patient’s age (3 weeks), serum IgG predominantly reflected maternally transferred IgG and therefore could not be used to reliably assess endogenous IgG production. The combination of clinical and immunological findings raised the suspicion of an Omenn syndrome, a phenotype frequently associated with hypomorphic defects in genes involved in V(D)J recombination and lymphocyte development (29).

**Figure 2:**
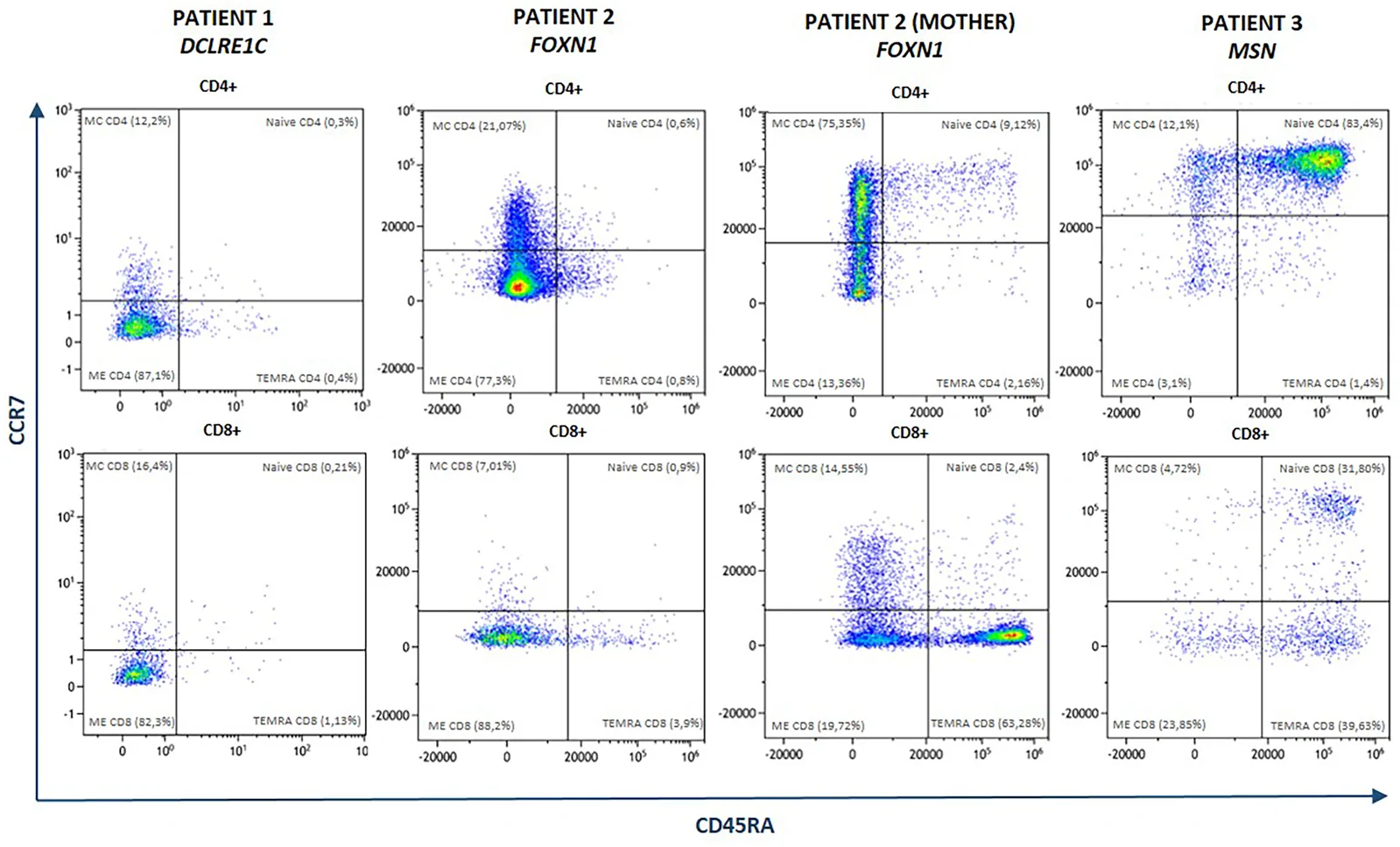
Flow cytometry analysis of the T-cell compartment in the three representative cases with abnormal TREC-based newborn screening. CD4^+^ and CD8^+^ T-cells subpopulations were analyzed in whole blood. All percentages are relative to the total CD4^+^ or CD8^+^ T-cells. Gating strategies define: naïve (CD45RA^+^CCR7^+^), central memory (CD45RA^-^CCR7^+^), effector memory (CD45RA^-^CCR7^-^) and terminally differentiated effector memory (TEMRA; CD45RA^+^CCR7^-^). Individual plots correspond to Patient 1 (DCLRE1C), Patient 2 (FOXN1), Patients 2’s mother (FOXN1) and Patient 3 (MSN).

**Table 2:** TRECs, immunophenotype and humoral evaluation of the patients.

Parametres	Patient 1 (*DCLRE1C*)	Patient 2 (*FOXN1*)	Patient 2 mother (*FOXN1*)	Patient 3 (*MSN*)	Pediatric reference values (1 month–2 years)†	Reference values (adults)
**TRECs, copies/punch**	**0/0**	**0/0**	N.D.	**11/14**	>30‡	N.D.
Lymphocyte populations						
Total lymphocytes, cells/µL	**1830**	**803**	1502	**542**	3260–8840†	1200–3000
CD3+, % (cells/µL)	**38 (689)**	**6 (48)**	**79** (1184)	**40 (217)**	52–77% (1850–5960)†	68–76% (850–2250)
CD4+, % (cells/µL)	**26 (481)**	**3 (24)**	**32 (483)**	32 **(173)**	30–58% (1140–3800)†	54–69% (500–1450)
CD8+, % (cells/µL)	**9 (165)**	**2 (16)**	**44** (663)	**8 (41)**	12–27% (540–1970)†	25–39% (160–950)
CD19+, % (cells/µL)	**0.1 (1)**	**42 (337)**	8.1 (122)	**4.9 (27)**	15–28% (640–1960)†	8–14% (100–500)
CD16+CD56+CD3−NK, % (cells/µL)	**53** (977)	**49** (400)	11 (169)	**52** (281)	3–24% (150–1330)†	4–20% (60–450)
T-cell receptor distribution (% of T cells)						
TCRαβ+	**81**	**53**	97	96	85–99§	85–99§
TCRγδ+	**19**	**46**	3	4	1–15§	2.9–5.5§
CD3+TCRαβ+CD4−CD8− (DNT)	0.8	2.4	0.9	0.8	0–3.5§	0–3.5§
CD4+ T-cell differentiation (% of CD4+ T cells)						
CD45RA+CD31+ (RTE)	**0**	**0.25**	**3.1**	65.34	**47–79†**	37–62
CD45RA+CCR7+ (naïve)	**0.3**	**0.6**	**9.1**	**83.4**	**54–80†**	39–53.7
CD45RA−CCR7+ (CM)	12.2	21.0	**75.3**	12.1	**10–26†**	30.7–40.4
CD45RA−CCR7− (EM)	**87.1**	**77.0**	13.1	3.1	**3–16†**	7.2–15.3
CD45RA+CCR7− (TEMRA)	**0.4**	**0.8**	2.1	**1.4**	**3–12†**	0.2–2.8
CD8+ T-cell differentiation (% of CD8+ T cells)						
CD45RA+CCR7+ (naïve)	**0.2**	**0.9**	**2.5**	**32**	**34–73†**	29.2–65.9
CD45RA−CCR7+ (CM)	**16.4**	7.0	**14.5**	4.7	**3–15†**	6.2–14.1
CD45RA−CCR7− (EM)	**82.3**	**88.0**	19.0	23.8	**9–47†**	16.6–34.6
CD45RA+CCR7− (TEMRA)	**1.1**	**3.9**	**63.3**	**39.8**	**7–25†**	5.9–38.1
Additional T-cell parameters						
CD4/CD8 ratio	2.9	1.5	**0.7**	**4.3**	1-3§	1.1–2.6
CD3+HLA-DR+, %	**69**	**87**	**50.7**	5	0-15§	0–28.6
CD4+HLA-DR+, %	**24**	**85.4**	**16.3**	4.49	0-15§	4.1–12.7
CD8+HLA-DR+, %	**65**	**93.1**	**79.0**	3.35	0-15§	9.1–44.6
Humoral immunity						
IgG, mg/dL	**305**	**1422**	1131	444	330–1160	700–1600
IgA, mg/dL	**<6.67**	**16**	**301**	51	35–242	40–230
IgM, mg/dL	<4	**259**	105	26	0–83	70–400

Values for major lymphocyte populations are expressed as percentage of total lymphocytes (absolute count, cells/µL). T-cell differentiation subsets and RTEs are expressed as percentages of their respective parent T-cell populations. †Pediatric reference intervals correspond to the 10th–90th percentiles reported for healthy children aged 1 month–2 years by García-Prat et al. (2019) (27). ‡TREC reference value corresponds to the locally established newborn-screening cutoff. §Reference interval obtained from our laboratory reference range and not from García-Prat et al. Adult reference intervals were derived from age- and sex-stratified reference values established using the standardized ONE-Study protocol (28). Abnormal patient values are shown in bold. DNT, double-negative T cells; RTE, recent thymic emigrants; CM, central memory; EM, effector memory; TEMRA, terminally differentiated effector memory T cells re-expressing CD45RA; N.D., not determined.

#### Genetic findings

Targeted NGS based on amplicons initially did not identify a causative molecular defect but revealed a marked loss of coverage affecting exons 7 and 8 of *DCLRE1C* (**Figure 3A**, top panel). Given that this gene was a strong candidate and additional molecular studies were undertaken. Sanger sequencing of the poorly covered region identified the c.488A>G (p.Tyr163Cys) variant, which was initially interpreted as heterozygous. However, this result was not consistent with either the severity of the phenotype or the expected autosomal recessive inheritance pattern of Artemis deficiency. Segregation analysis demonstrated the presence of the same variant in both parents and revealed an atypical allelic distribution, prompting re-evaluation of the sequencing strategy (**Figures 3B**, left). Further investigation identified interference from the highly homologous *DCLRE1CP1* pseudogene, which affected the sequencing within the genomic region. Selective gene amplification was achieved using locus-specific primers targeting the 5% non-homologous region to exclude pseudogene interference. Finally, the c.488A>G (p.Tyr163Cys) variant was confirmed in homozygosity in the patient and in heterozygosity in both parents (**Figures 3B**, right). According to the American College of Medical Genetics and Genomics/Association for Molecular Pathology (ACMG/AMP) guidelines (23), the variant was classified as likely pathogenic and is not described in ClinVar (GenBank accession number PZ603458). These findings established the molecular diagnosis of Artemis deficiency and resolved the discrepancy between the initial genetic results and the patient’s clinical and immunological phenotype (**Table 3**).

**Figure 3:**
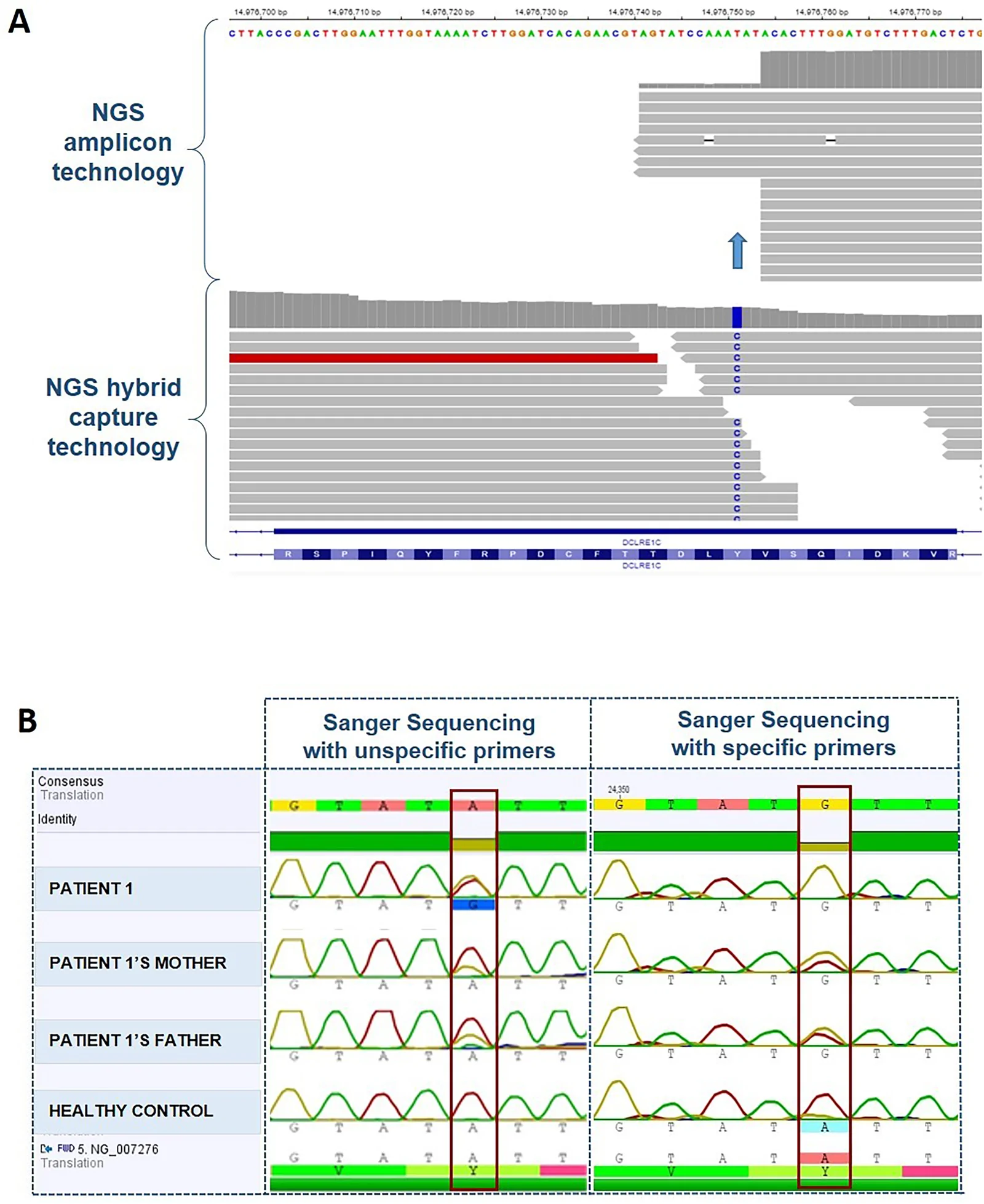
Pseudogene interference leading to misinterpretation of the pathogenic *DCLRE1C* variant. **(A)** Integrative Genomics Viewer (IGV) alignment of next-generation sequencing coverage across the *DCLRE1C* locus using amplicon-based technology (top) and hybrid-capture technology (bottom). The blue arrow indicates the position of the mutation p.Tyr163Cys. **(B)** Sanger sequencing chromatograms illustrating the effect of pseudogene interference on variant interpretation. Sequencing region performed with unspecific primers (left) and specific primers (right) in Patient 1, Patient 1’s mother, Patient 1’s father, and a healthy control. Red boxes indicate the position of the nucleotide substitution.

**Table 3:** Genetic findings in the described patients*.

Patient ID	Gene	cDNA	Protein	Consequence	Zygosity	VAF (%)	Inheritance	ACMG	ACMG CRITERIA
P1	*DCLRE1C*	c.488A>G	p.Tyr163Cys	Missense	Homozygous	100	AR	LP	PM2 (Supporting) PM3 (Moderate) PP4 (Moderate)
P2	*FOXN1*	c.1465del	p.Gln489ArgfsTer61	Frameshift	Heterozygous	44.8	AD†	P	PVS1 (Very Strong) PS4_(Moderate) PM2 (Supporting) PM6 (Supporting) PP4 (Moderate)
P3	*MSN*	c.511C>T	p.Arg171Trp	Missense	Hemizygous	100	XL	P	PS1 (Strong) PP5 (Moderate) PM2 (Supporting) PP3 (Moderate) BP1 (Supporting)

*Variants are described according to Human Genome Variation Society (HGVS) nomenclature. Variant pathogenicity was assessed according to ACMG/AMP criteria, incorporating gene-specific ClinGen Variant Curation Expert Panel (VCEP) specifications when available (https://erepo.clinicalgenome.org/) (23). †Monoallelic *FOXN1* variants show semidominant inheritance with variable expressivity. Abbreviations: VAF, variant allele frequency; AR, autosomal recessive; XL, X-linked; LP, likely pathogenic; P, pathogenic; PVS, pathogenic very strong; PS, pathogenic strong; PM, pathogenic moderate; PP, pathogenic supporting.

#### Therapeutic intervention and outcome

Following diagnosis, the patient received antimicrobial prophylaxis, immunoglobulin replacement therapy, nutritional support, and immunosuppressive treatment with cyclosporine for control of inflammatory manifestations. A fully HLA-matched sibling donor was identified and the patient underwent allogeneic hematopoietic stem cell transplantation (HSCT) using a reduced-intensity conditioning regimen based on fludarabine and treosulfan. At day +100 post-transplantation, immunological evaluation demonstrated recovery of T-cell numbers, including CD4^+^, CD8^+^ and CD19^+^ subsets (data not shown), together with clinical improvement and resolution of the inflammatory manifestations. The patient achieved successful immune reconstitution and remains alive and well five years post-transplant. He has complete donor chimerism and has not developed acute or chronic graft-versus-host disease.

### Case 2. FOXN1 deficiency

#### Clinical and immunological findings

The patient is a 29 + 6 week female infant referred due to absent TRECs on her NBS (0/0 copies per punch). During the first month of life, she developed generalized erythroderma (prior to any blood transfusion) together with generalized lymphadenopathy. Immunological evaluation demonstrated severe lymphopenia (803 cells/µL) with markedly reduced T and B lymphocytes (48 cells/µL and 337 cells/µL, respectively), CD4^+^ (24 cells/µL) and CD8^+^ (16 cells/µL) T cells. NK cells represented the predominant circulating lymphocyte population (400 cells/µL). T-cell compartment studies showed profoundly impaired thymic output, evidenced by a severe reduction of recent thymic emigrants (0.25%) and naïve CD4^+^ and CD8+ T cells (0.6% and 0.9%, respectively) (**Figure 2**). Effector-memory T cells predominated in both CD4^+^ (77%) and CD8^+^ (88%) compartments. T-cell activation was markedly increased, with HLA-DR expression detected in 85.4% of CD4^+^ and 93.1% of CD8^+^ T-lymphocytes. Serum immunoglobulin analysis showed elevated IgG (1422 mg/dL) and IgM (259 mg/dL) concentrations (**Table 2**).

The family history of the patient’s mother showed persistent clinical and immunological abnormalities. She presented with mild ectodermal features, including sparse hair and nail dystrophy, together with decreased CD4^+^ T-cell counts (483 cells/µL) and an altered maturation profile characterized by low frequencies of recent thymic emigrants (3.1%) and naïve CD4^+^ and CD8^+^ T cells (9.1% and 2.5%, respectively) (**Figure 2**). CD8^+^ T-lymphocytes presented an activated phenotype (CD8^+^HLA-DR^+^: 79%) (**Table 2**).

#### Genetic findings

Whole-exome sequencing identified a germline heterozygous frameshift variant in *FOXN1*: c.1465del (p.Gln489ArgfsTer61). Segregation analysis confirmed that the variant was inherited from the patient’s mother. According to the ACMG/AMP criteria (23), the variant was classified as pathogenic, establishing the diagnosis of monoallelic FOXN1 deficiency. This variant and the associated phenotype are consistent with previous reports that describe severe neonatal T-cell lymphopenia with variable intrafamilial expressivity in FOXN1 deficiency (30, 31)(**Table 3**).

#### Therapeutic intervention and outcome

The presence of the same pathogenic variant in the mother, who exhibited a significantly milder clinical phenotype and detectable naïve T-cell populations, supported conservative management (31). Accordingly, the patient received supportive care and underwent serial clinical and immunological monitoring. Follow-up evaluations revealed persistent immune abnormalities. However, given the thymic stromal nature of the defect, the family history, and the limited experience with this condition in the setting of NBS, close multidisciplinary surveillance was planned (32).

### Case 3. Moesin deficiency

#### Clinical and immunological findings

The patient is a male infant referred following an abnormal NBS result that showed persistently reduced TRECs (11/14 copies per punch). At 20 days of age, the patient was clinically healthy, but immunological evaluation demonstrated severe lymphopenia (542 cells/µL) highly suggestive of leaky SCID. Immunophenotyping revealed marked reductions in T and B lymphocytes (217 cells/µL and 27 cells/µL, respectively), including CD4^+^ (173 cells/µL) and CD8^+^ (41 cells/µL) T cells. Despite the low absolute lymphocyte counts, T-cell compartment analysis demonstrated a preserved proportion of naïve CD4+ T cells (83.4%) and, to a lesser extent, naïve CD8+ T cells (32%) (**Figure 2**). Importantly, although the proportion of naïve CD4+ T cells was within the normal range before treatment, their absolute number remained reduced in the context of severe T-cell lymphopenia. Recent thymic emigrants represented 65.3% of CD4+ T cells. No increase in T-cell activation markers was observed (**Table 2**).

#### Genetic findings

Targeted next-generation sequencing identified a hemizygous missense variant in *MSN*: c.511C>T (p.Arg171Trp). The variant was classified as pathogenic according to the ACMG/AMP criteria (23), establishing the diagnosis of X-linked moesin deficiency. This variant has previously been reported in patients with *MSN*-associated immunodeficiency (33, 34) (**Table 3**).

#### Therapeutic intervention and outcome

Following diagnosis, the patient underwent clinical and immunological follow-up. Serial evaluations confirmed persistent lymphopenia, despite preservation of the relative proportion of naïve T-cell subsets and recent thymic emigrants. The patient is under surveillance for infectious complications and immune dysregulation associated with moesin deficiency.

Therefore, during the first 11 months following the implementation of SCID newborn screening (NBS) in the Autonomous Communities of La Rioja, Aragón and Navarra (Spain), 12,388 newborns were screened. Through this program, two patients (P2 and P3) were identified with low TRECs levels and an initial suspicion of SCID, who were ultimately diagnosed with non-SCID conditions.

## Discussion

NBS based on TRECs quantification has fundamentally transformed the diagnosis and management of SCID by enabling identification of affected newborns before the onset of life-threatening infections. Following an abnormal TRECs result, prompt confirmation in specialized centers with expertise in IEI is essential. Clinical and immunological evaluation, including rapid assessment, T-, B- and NK-cell immunophenotyping, characterization of naïve and memory T-cell compartments, quantification of RTEs, and early molecular testing, enables discrimination between distinct biological mechanisms (7, 9, 14, 15, 25, 26, 35, 36). The ultimate goal is to translate these biological findings into an individualized therapeutic strategy, where multidisciplinary evaluation can be performed with the speed and expertise required for these time-critical conditions. While classical forms of SCID require urgent HSCT or, for selected disorders, gene or enzyme replacement therapy, other causes of non-SCID T-cell lymphopenia cannot benefit from HSCT because the primary defect resides outside the hematopoietic compartment (13).

The three patients presented here exemplify how abnormal TRECs results require markedly different diagnostic pathways and therapeutic approaches depending on the underlying mechanism of impaired thymic output. Although these entities converge on a common biological endpoint (reduced or absent thymic export of naïve T cells), their molecular basis, clinical evolution and therapeutic implications differ profoundly (**Figure 1**) (7, 9). The case of Artemis deficiency represents a canonical defect in antigen receptor recombination and DNA repair. Artemis, encoded by *DCLRE1C*, is a critical component of the non-homologous end joining (NHEJ) pathway required for repair of DNA double-strand breaks generated during V(D)J recombination (20). Successful T-cell receptor rearrangement is a prerequisite for thymocyte maturation and TRECs generation; consequently, failure of recombination results in absent or profoundly reduced TRECs, reflecting an early developmental arrest (**Figure 1**) (7). Nevertheless, the principal diagnostic challenge in this patient arose from technical limitations of the initial molecular analysis rather than the biological complexity. The lack of coverage for exons 7 and 8 of the *DCLRE1C* gene in the amplicon NGS panel (**Figure 3A**, top panel) was due to the high sequence homology (95%) with the *DCLRE1CP1* pseudogene, where sequence variations within short primer-binding sites can cause amplification failure (primer drop-out) (37, 38). This technical limitation initially led to an incorrect heterozygous interpretation of the variant (**Figure 3B**, left panel) despite an immunological phenotype highly suggestive of Artemis deficiency. Subsequent locus-specific Sanger sequencing resolved the diagnosis (39, 40) (**Figure 3B**, right panel). However, retrospective analysis using a hybrid-capture-based panel successfully overcame these limitations, providing robust and uniform coverage across these critical *DCLRE1C* exons (**Figure 3A**, bottom panel). The long hybridization probes utilized in capture methods tolerate nucleotide mismatches without compromising binding efficiency, which combined with a multi-probe strategy and paired-end sequencing, optimizes mapping precision and specificity (41). Consequently, the hybrid-capture panel correctly identified the pathogenic variant in a homozygous state. This case highlights the importance of integrating molecular findings with clinical and immunological data, particularly when the genetic results appear discordant with the biological phenotype. Such delays could have severe clinical consequences in Artemis deficiency, where early diagnosis is essential to optimize HSCT and adapt conditioning regimens to avoid the characteristic radiosensitivity of these patients (36).

The second case represents a distinct mechanistic category where the primary defect resides within the thymic stromal microenvironment rather than the hematopoietic compartment (**Figure 1**). FOXN1 encodes a transcription factor indispensable for the differentiation, maintenance, and function of thymic epithelial cells (TECs), which provide the structural and molecular framework required for normal thymocyte development (42, 43). Cortical and medullary TECs orchestrate multiple stages of T-cell ontogeny by regulating Notch signaling, IL-7 production, thymocyte migration, positive and negative selection, and establishment of central tolerance. Consequently, impairment of FOXN1 primarily alters the thymic niche while leaving hematopoietic progenitors intrinsically intact (32). TRECs in this case, indicates failure of the thymic microenvironment to adequately support T-cell maturation. This distinction is clinically relevant because biologically similar TRECs results may arise from fundamentally different pathogenic mechanisms requiring different therapeutic strategies (13, 30, 31, 44, 45). This patient further illustrates the remarkable phenotypic heterogeneity associated with monoallelic FOXN1 deficiency. While the proband presented with profound neonatal T-cell lymphopenia, absent TRECs and an Omenn-like phenotype characterized by marked T-cell activation and expansion of effector T-cell populations, the patient’s mother, who carried the same pathogenic variant, exhibited a significantly milder clinical presentation but with persistent immunological abnormalities. This intrafamilial variability is consistent with previous reports demonstrating incomplete penetrance, variable expressivity, and age-dependent immunological evolution in monoallelic FOXN1 deficiency (30, 31). The persistence of detectable naïve T cells and RTEs in the mother indicates preservation of residual thymic function that contrasts with the marked neonatal thymopoiesis impairment observed in her preterm child. Furthermore, the prematurity is a relevant confounding factor in the interpretation of NBS, since TRECs levels are lower in preterm infants and increase progressively with gestational age. However, TRECs have been reported to increase by approximately 9.6% for each additional week of gestation (46). In P2, TRECs remained persistently absent. This finding, together with the immunological phenotype (T-cell lymphopenia and a markedly reduced naïve T-cell compartment) supports that the NBS result represented a true abnormality of thymic output rather than being attributable to prematurity alone. These observations have direct therapeutic implications, unlike classical forms of SCID, HSCT alone may not fully correct FOXN1 deficiency because the primary defect resides within the thymic epithelial compartment rather than in hematopoietic cells. Therefore, treatment decisions should be guided by the severity of thymic dysfunction (47)(**Figure 1**). In severe congenital athymia, thymic transplantation represents the preferred definitive therapy, whereas patients with residual thymic function and progressive spontaneous immune maturation are managed conservatively with close clinical and immunological follow-up (48). In this patient, the demonstration of residual thymopoiesis together with the relatively mild phenotype observed in the affected mother supported a conservative management strategy with longitudinal monitoring rather than immediate HSCT (49). A limitation of the study is that maternal T-cell engraftment was not evaluated in P2, which could contribute to the observed erythroderma and activated T-cell phenotype. However, the diagnosis of FOXN1 deficiency remains supported by genetic testing and familial segregation, so maternal engraftment would not contribute for the primary thymic defect.

The third case illustrates a fundamentally different mechanism affecting immune-related actinopathies rather than early thymocyte development (50). Moesin, a member of the ezrin-radixin-moesin (ERM) family, links the plasma membrane to the actin cytoskeleton and plays a central role in lymphocyte adhesion, migration, immunological synapse formation, intracellular signaling and cellular survival. In moesin deficiencies, V(D)J recombination remains intact, allowing T-cell receptor rearrangement. Reduced TRECs reflect impaired cellular competence, altered thymocyte maturation efficiency or abnormalities in peripheral lymphocyte maintenance rather than a failure of receptor generation itself (33, 34). This biological distinction highlights that reduced TRECs values are not exclusively associated with defects affecting early T-cell development but may also result from abnormalities occurring at later stages of lymphocyte maturation and maintenance (7). Identification of Moesin deficiency through TREC-based newborn screening has been reported but remains uncommon. Delmonte et al. described the first case of X-linked moesin-associated immunodeficiency identified through SCID newborn screening, notably carrying the same recurrent c.511C>T (p.Arg171Trp) variant identified in our patient (33). Our case provides an additional example of early detection through TRECs screening and illustrates that reduced TRECs and severe neonatal lymphopenia may coexist with relative preservation of naïve T cells and recent thymic emigrants. Immunophenotypic findings associated with moesin deficiency are highly heterogeneous, ranging from isolated T-cell lymphopenia to combined immunodeficiency with progressive immune dysregulation and depletion of naïve T-cell populations (33, 34). The reduced counts of RTEs and naïve T-cell subsets observed in this patient suggest that impaired peripheral T-cell maintenance may contribute to an abnormal NBS result despite preserved early thymic differentiation (11, 51).Taken together, these cases delineate a hierarchical model of T-cell development involving three distinct biological levels: antigen receptor recombination and DNA repair, thymic stromal support and lymphocyte cytoskeletal regulation and immune homeostasis. Reduced TRECs should instead be interpreted as a quantitative biomarker of impaired thymic output requiring additional mechanistic dissection. Therapeutic decisions should be guided by the affected biological compartment, while hematopoietic defects such as Artemis deficiency or other SCID conditions are appropriately treated with HSCT or gene therapy (15, 36), thymic microenvironment disorders, including FOXN1 deficiency, may require alternative approaches such as thymic transplantation or conservative follow-up depending on residual thymic function. Likewise, disorders primarily affecting lymphocyte homeostasis, such as moesin deficiency, require individualized management based on their clinical and immunological evolution (34).

Ultimately, our findings reinforce the need for a multidimensional diagnostic approach that integrates NBS results with detailed immunophenotyping, functional and genetic studies in specialized clinical, immunological and HSCT units. Such an integrative strategy is essential to achieve accurate diagnosis and support mechanism-based therapeutic decisions in patients with IEI.

## Data Availability

The datasets presented in this study can be found in online repositories. The names of the repository/repositories and accession number(s) can be found below: https://www.ncbi.nlm.nih.gov/genbank/, PZ603458.
